# CD200 genotype is associated with clinical outcome of patients with multiple myeloma

**DOI:** 10.3389/fimmu.2024.1252445

**Published:** 2024-02-22

**Authors:** Yolanda Gonzalez-Montes, Gemma Osca-Gelis, Rocío Rodriguez-Romanos, Alicia Villavicencio, Marta González-Bártulos, Francesca Llopis, Victòria Clapes, Albert Oriol, Anna Sureda, Lourdes Escoda, Josep Sarrà, Ana Garzó, Natàlia Lloveras, Beatriz Gómez, Isabel Granada, David Gallardo

**Affiliations:** ^1^ Hematology Department, Institut Català d’Oncologia, Hospital Dr. Josep Trueta, Institut d’Investigació Biomèdica de Girona (IDIBGI), Josep Carreras Research Institute, Universitat de Girona, Girona, Spain; ^2^ Hospital Cancer Registry Unit, Catalan Institute of Oncology, Girona, Spain; ^3^ Research Group on Statistics, Econometrics and Health (GRECS), Universitat de Girona, Girona, Spain; ^4^ Center CIBER of Epidemiology and Public Health (CIBERESP), Girona, Spain; ^5^ Clinical Hematology Department, Institut Català d’Oncologia, L’Hospitalet de Llobregat, Institut d’Investigaciò Biomèdica de Bellvitge (IDIBELL), Universitat de Barcelona, Barcelona, Spain; ^6^ Hematology Department, Institut Català d’Oncologia, Hospital Germans Trias i Pujol, Josep Carreras Research Institute, Barcelona, Spain; ^7^ Hematology Department, Institut Català d’Oncologia, Hospital Joan XXIII, Universitat Rovira i Virgili (URV), Tarragona, Spain

**Keywords:** CD200 polymorphisms, multiple myeloma, immune checkpoint, bone marrow microenvironment, immune disfunction

## Abstract

Immune dysfunction in patients with MM affects both the innate and adaptive immune system. Molecules involved in the immune response pathways are essential to determine the ability of cancer cells to escape from the immune system surveillance. However, few data are available concerning the role of immune checkpoint molecules in predicting the myeloma control and immunological scape as mechanism of disease progression. We retrospectively analyzed the clinical impact of the CD200 genotype (rs1131199 and rs2272022) in 291 patients with newly diagnosed MM. Patients with a CD200 rs1131199 GG genotype showed a median overall survival (OS) significantly lower than those with CC+CG genotype (67.8 months versus 94.4 months respectively; p: 0.022) maintaining significance in the multivariate analysis. This effect was specially detected in patients not receiving an autologous stem cell transplant (auto-SCT) (p < 0.001). In these patients the rs1131199 GG genotype negatively influenced in the mortality not related with the progression of MM (p: 0.02) mainly due to infections events.

## Introduction

Multiple myeloma (MM) is a hematological malignancy that is characterized by clonal proliferation of malignant plasma cells in the bone marrow, monoclonal protein in the blood or urine and associated organ dysfunction. Progressive immune impairment is a characteristic of MM development, allowing neoplastic plasma cells to escape from immune surveillance, promoting disease growth and resistance to therapy (1). The pathogenesis of immune dysfunction and neoplastic evasion in MM is facilitated by multiple cytokine and cellular signaling pathways, which decrease immune effector cell function and determine a suppressive bone marrow microenvironment (2). The continued development of news therapies have improved the outcome of MM patients, but unfortunately it remains an incurable disease and the relapse is common due to residual, drug-resistant, myeloma cells that survive to the treatment (3).

Immunological scape of cancer cells is a recognized mechanism of disease progression. Several factors have been implicated in the evasion of cancer cells from immune surveillance, there is evidence that malignant cells can enhance the expression of inhibitory immune checkpoint molecules to avoid immune recognition and elimination (4). CD200 is a type-1 membrane glycoprotein of the immunoglobulin supergene family, related structurally to the B7 family of co-stimulatory receptors, which is expressed on several cell types relevant to the inflammatory and immune cascade, such as resting dendritic cells, thymocytes, endothelial cells, neurons and osteoblast precursors, as well as by activated B and T cells (5). CD200 acts as an immune checkpoint molecule through its receptor CD200R, which is expressed mainly in myeloid cells such as monocytes, macrophages, dendritic cells, mast-cells, as well as B lymphocytes and a subset of T cells (6, 7). Similar to other immune checkpoint molecules, CD200 plays an important regulatory role in control of autoimmune diseases, infection, allergy, transplantation and cancer. CD200 modulates immune cell activity by inhibiting secretion of pro-inflammatory cytokines (IL2 and interferon-gamma), enhancing anti-inflammatory cytokine secretion (IL-10 and IL-4) (7), increasing production of myeloid-derived suppressor cells (8) and T regulatory cells (9) and suppressing the activities of both natural Killer cells (10) and basophils (11), leading to an impaired antitumor activity (12, 13). High CD200 expression has been found to be expressed on several types of cancer, including several hematological neoplasms (14), malignant melanoma (15) and neuroendocrine tumors (16).

A major role in the development of the immunosuppressive state in MM patients has been attributed to an increased expression of immune checkpoint molecules that negatively regulate T-cell function, such as PDCD1, CTLA4, BTLA and T-cell immunoglobulin and ITIM domains (TIGIT) on T cells (17–19). In addition, several studies have shown a relationship between genetic polymorphisms in co-stimulatory/inhibitory molecules and susceptibility to the development MM (20–24). However, few date are available concerning the relevance of immune checkpoint molecules in the kinetics of progression of MM patients. Recently, our group showed that the CTLA4 genotype may be useful to identify MM patients with high risk of progression (25).

Although previous studies have reported the correlation between CD200 expression level in MM cells and survival (26–28), there are not data concerning the potential impact of genetic polymorphisms in the CD200 gene on clinical outcome in patients with MM. In our study we intend to evaluate whether the presence of genetic variations within this checkpoint molecule are associated with an increased risk of progression in patients with MM.

## Patients and methods

We retrospectively analyzed 291 patients with newly diagnosed MM who were eligible for first-line treatment and followed at the Catalan Institute of Oncology centers between 1995 and 2020. DNA was obtained from peripheral blood or bone marrow samples at different stages of the disease. Biological samples and clinical data were processed following standard operating procedures and approved by the Ethics and Scientific Committees. All patients signed an informed consent and the study met with the recommendations of the Helsinki declaration. Samples and data from patients included in this study were provided by the IDIBGI Biobank (Biobanc IDIBGI, B.0000872), integrated in the Spanish National Biobanks Network and they were processed following standard operating procedures with the appropriate approval of the Ethics and Scientific Committees. Clinical characteristics of patients and first-line treatments are summarized in **Table 1**. The median follow up was 51.4 months for these patients.

**Table 1:** Clinical characteristics of patients with MM from 1995-2020.

Characteristics (N=291)	Total *%^a^*
Age (years)	
Median (range)	66 (59–73)
Sex	
Men	56.0
Women	44.0
Type of Monoclonal protein	
IgG	52.4
IgA	27.8
Light chains	14.5
Others	5.2
History of MGUS	9.4
ISS stage	
I-II	70.0
III	30.0
LDH	
High	12.0
Normal	88.0
^†^Kidney failure	
No	76.5
Yes	23.5
HSCT	
Yes	42.5
No	57.5
First Line Therapy	
Alkylating agents	18.6
Proteasome inhibitors	48.9
Immunomodulatory drugs	5.6
Proteasome Inhibitors + Immunomodulatory drugs	26.5
Daratumumab monotherapy	0.4

^a^Except where specified.

MGUS, monoclonal gammopathy of undetermined significance. **^†^** Kidney failure, creatinine ≥ 2 mg/dl. HSCT, hematopoietic stem cell transplantation. LDH, lactate dehydrogenase.

In addition, during the same period of time and in the same participant centers a second cohort of 67 patients with newly diagnosis of smoldering MM was specifically analyzed to correlate the genotype in the studied polymorphisms with the time to progression to symptomatic MM. Clinical characteristics of patients are summarized in **Supplementary Table S1**.

### Expression of CD200 in plasma cell and lymphocyte subsets populations

The immunophenotypic studies were performed using fresh bone aspiration samples in EDTA from patients with newly diagnosed MM. A standard protocol of stain-lyse-wash was performed using BC Versalyse™ and fixed with paraformaldehyde, stained with the following 9 fluorescence panel: CD38 PB- CD45-KrO, CD81-FITC, CD27-PE, CD117-ECD, CD19-PC5.5, CD200-PC7 (clone OX-104: IGg1 against Human OX2CD4d3 + 4 soluble fusion protein), CD138-APC, CD56-APC-AF750. Acquisition was performed with 3 laser Beckman Coulter (BC) Navios Flow Cytometer, calibrated everyday with BC Flowset Pro particles with a minimal acquisition of 100.000 events. FCS analysis was performed using a Batch process with BC Kaluza software with a predesigned template to standardize measures. Percentage of positive expression, median fluorescence intensity, variation coefficient and pattern of expression was assessed in different populations: normal and abnormal plasma cell compartment, B mature lymphocytes CD27-, B mature lymphocytes CD27+, T lymphocytes CD27+ CD19- CD56-, and NK lymphocytes. Additionally, the median fluorescence intensity ratio (MFIR) of CD200 was calculated between the abnormal plasma cell and the lymphocyte B CD27 negative.

### Genotype analysis

DNA was extracted from 200µl of whole blood using a QIAamp DNA Blood Mini Kit (Qiagen, GmbH, Hilden, Germany) according to the manufacturer’s instructions and stored at -80°C until use.

We analyzed two polymorphisms of CD200 gene: rs1131199 and rs2272022. The genotype for these polymorphisms was determined via allelic discrimination plots on Applied Biosystems™ QuantStudio™ 7 Flex Real-Time PCR System by using TaqMan^®^ SNP Genotyping Assays real time PCR according to the manufacturer’s instruction.

### Statistical analyses

Allele frequencies and genotypes were formulated by direct counting. Homogeneity between genotype groups was evaluated using the chi-square test or Fisher’s exact test for qualitative variables and Student’s test for continuous variables. Kaplan-Meier curves were obtained to determine overall survival (OS) and progression free survival (PFS) and curves were compared using the log-rank test. A two-sided p value of 0.05 or lower was considered to be statistically significant. Cumulative incidence considering competitive risks was determined to assess the relationship between the genetic groups and the time to progression for patients with symptomatic MM receiving first line of treatment. Comparison of curves was made by Gray’s Test. Multivariate analysis was performed using the Cox regression model. All the variables with a p value at or below 0.2 in the univariate analysis were included in the multivariate analysis.

## Results

### CD200 genotype distribution

CD200 rs1131199 genotype could be successfully determined in 289 analyzed patients. The G allele was the majoritarian, being detected in 78.9% of cases whereas the C allele was identified in 69.9% of the studied cases. The genotype distribution showed 87 patients (30.1%) homozygous for the G allele, 61 homozygous for the C allele (21.1%) and 141 heterozygous CG (48.8%).

CD200 rs2272022 genotype could be successfully determined in 279 analyzed patients. The C allele was the majoritarian, being detected in 82.4% of cases whereas the A allele was identified in 63.8% of the studied cases. The genotype distribution showed 101 patients (36.2%) homozygous for the C allele, 49 homozygous for the A allele (17.6%) and 129 heterozygous AC (46.2%).

**Table 2** shows the genotypes distribution for each analyzed polymorphism. The genotype frequencies were comparable to the previously described in Caucasian population.

**Table 2:** Frequencies of genetics polymorphisms in the analyzed patients.

Genes	Genotype	%
CD200 rs1131199	CC	21.1
	GG	30.1
	CG	48.8
CD200 rs2272022	AA	17.6
	CC	36.2
	AC	46.2

### Homogeneity between genotype groups

The comparison of clinical prognostic factors at diagnosis between genetic groups for each polymorphism showed a balanced distribution for age, sex, type of monoclonal protein, former history of monoclonal gammopathy of unknown significance (MGUS), International Staging System (ISS), cytogenetics, serum LDH levels or kidney failure. Moreover, the proportion of patients receiving an autologous peripheral blood stem cell transplant (PBSCT) was also comparable within genetic groups. **Table 3** shows the comparison of clinical characteristics of the symptomatic MM patients according to the CD200 genotypes.

**Table 3:** Patient`s characteristics according to CD200 rs1131199 and CD200 rs2272022.

Characteristics	Total	CD200 rs1131199			CD200 rs2272022		
	*%*	CC+CG	GG	*P*	AA+AC	CC	*P*
Total	291 (100.0)	202 (69.9)	87 (30.1)		178 (63.8)	101 (36.2)	
Age (years)							
Median (range)	66 (59–73)	65 (59–72)	67 (60–76)	0.169	65 (59–72)	67 (60–76)	0.739
Sex							
Men	56.0	56.9	54.0	0.648	55.6	58.4	0.650
Women	44.0	43.1	46.0		44.4	41.6	
Age groups							
< 66 years	49.5	51.0	45.9	0.429	47.8	51.5	0.548
>= 66 years	50.5	49.0	54.1		52.2	48.5	
Type of Monoclonal protein							
IgG	52.4	55.7	43.5	0.156	55.1	49.0	0.333
IgA	27.8	25.8	33.0		26.2	30.0	
Light chains	14.5	14.0	16.5		13.6	16.0	
Others	5.2	4.5	7.1		5.1	5.0	
History of MGUS	9.4	10.1	8.2	0.923	9.7	10.1	0.687
ISS stage (N= 229)							
I/II	70.0	71.4	66.2	0.413	72.5	67.4	0.398
III	30.0	28.6	33.8		27.5	32.6	
Cytogenetic profile (N=182)							
High-risk cytogenetic by FISH*	8.8	9.9	6.1	0.563	8.8	10.3	0.737
Standard risk	91.2	90.1	93.9		91.2	89.7	
LDH							
High	12.0	14.1	7.7	0.190	16.0	7.3	0.068
Normal	88.0	85.9	92.3		84.0	92.7	
Kidney failure							
No	76.5	77.2	74.4	0.616	76.3	77.1	0.889
Yes	23.5	22.8	25.6		23.7	22.9	
HSCT							
Yes	42.5 (auto)	43.1	41.9	0.841	42.2	45.0	0.652
No	57.5	56.9	58.1		57.8	55.0	

HSCT, hematopoietic stem cell transplantation. MM, multiple myeloma. MGUS, monoclonal gammopathy of undetermined significance. *FISH, del(17p), t(4,14), t(14,16). LDH, lactate dehydrogenase.

### Flow cytometry CD200 expression

As the expression of CD200 in MM cells has been previously correlated with worse prognosis, we explored whether the CD200 genotype influenced the intensity of CD200 expression on neoplastic plasm cells in a cohort of 47 patients. Median fluorescence intensity (MFI) of CD200 in neoplastic plasm cells was not different between genetic groups: 2.03 (interquartile range (IQR) 8.27) for patients with CD200 rs1131199 CC or CG genotypes and 3.31 (IQR 12.81) for patients with CD200 rs1131199 GG genotype (p: 0.541). The ratio of CD200 expression between neoplastic plasm cells and normal B lymphocytes was 1.17 (IQR: 3) *vs*. 3.16 (IQR: 3) (p: 0.109).

### Correlation between CD200 genotype and progression to symptomatic multiple myeloma

When analyzing the cohort of 67 patients with newly diagnosed smoldering MM, we did not find any correlation between the analyzed genotypes and the time to receive a first line of therapy. In this cohort, the median the time to receive therapy was 14.1 months for patients with CD200 rs1131199 GG genotype versus 30 months for those patients with the CC+GG genotype (p: 0.4). Similar results were obtained when analyzing the impact of the CD200 rs2272022 (AA+AC: 35.6 months *vs*. CC: 19.1 months; p: 0.3).

### CD200 genotype and clinical outcome after treatment of symptomatic multiple myeloma

**Figure 1:**
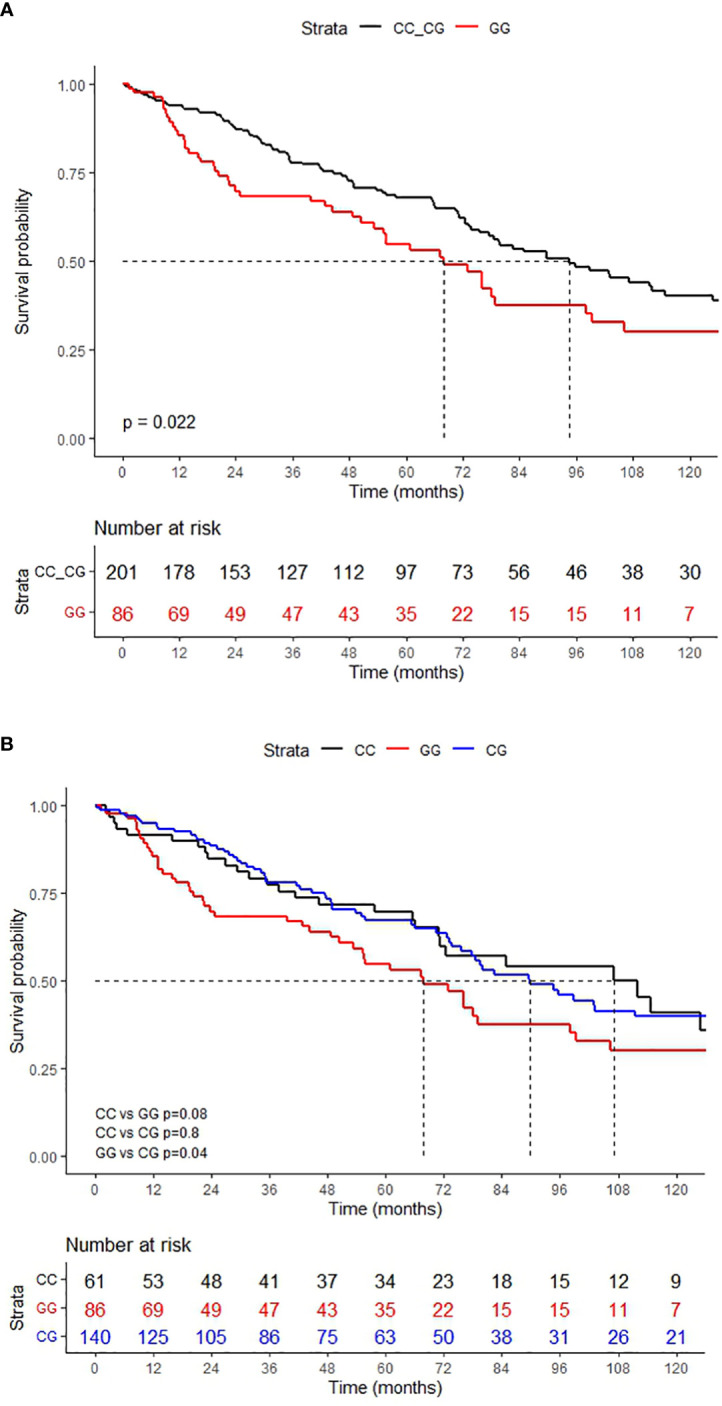
OS according to the CD200 rs1131199 genotype. **(A)** grouped genotypes and **(B)** independent genotypes.

Patients with a CD200 rs1131199 GG genotype showed a median OS significantly lower than those with CC or CG genotype: 67.8 months (95% confidence interval (CI): 53.2 – 99.0) and 94.4 months (95% CI: 77.5 – 125) respectively; p: 0.022) (**Figure 1**). The 5-year OS rate was 53.0% for patients with GG genotype *vs* 68.0% for patients with grouped CC and CG genotype. Age ≥ 66 years (67.1 *vs* 124.6 months; p < 0.001), high-risk cytogenetics (29.2 *vs* 98.6 months; p < 0.001), high ISS (55.2 *vs* 94.4 months; p: 0.001) and not receive a hematopoietic stem cell transplantation (66.0 *vs* 142.0 months; p < 0.001) were also risk factors for lower OS. In the multivariate analysis, the CD200 rs1131199 polymorphism remained as an independent risk factor for OS (Hazard Ratio (HR): 1.8; 95% CI: 1.1–3.2; p: 0.03). **Table 4** shows the results of the multivariate analysis. When the causes of death were analyzed according to the CD200 rs1131199 genotype the main raison was the progression of disease in both genetic groups (GG: 61.2% *vs* CC and CG pooled together: 55.2%), followed by infection (GG: 16.3% *vs* CC+CG: 15.6%) without detecting statistically significant differences between these groups (p: 0.914).

**Table 4:** Univariate and multivariate analysis.

	Univariate analysis				Multivariate analysis			
	OS HR (95% CI)	*p*	PFS HR (95% CI)	*p*	OS HR (95% CI)	*p*	PFS HR (95% CI)	*p*
Sex								
Men	1.3 (1.0-1.9)	0.09	1.4 (1.1-1.9)	0.01	1.1 (0.7-1.9)	0.660	1.5 (1.0-2.3)	0.070
Age group								
>= 66 years	2.4 (1.7-3.4)	<0.001	1.8 (1.4-2.4)	<0.001	1.4 (0.63-3.2)	0.417	1.1 (0.5-2.1)	0.900
Cytogenetic risk								
High	3.3 (1.6-6.8)	<0.001	2.0 (1.1-3.7)	0.03	3.4 (1.6-7.4)	0.002	1.9 (1.0-3.7)	0.006
ISS stage								
III	1.9 (1.3-2.8)	0.001	1.8 (1.3-2.4)	<0.001	2.1 (1.2-3.5)	0.007	1.8 (1.2-2.9)	0.007
HSCT								
Non-HSCT	2.4 (1.7-3.5)	<0.001	2.0 (1.5-2.7)	<0.001	1.8 (0.8-4.1)	0.185	1.8 (0.9-3.5)	0.110
Genotype CD200 rs1131199								
GG	1.5 (1.1-2.2)	0.02	1.1 (0.8-1.5)	0.42	1.8 (1.1-3.2)	0.030	1.0 (0.6-1.5)	0.820

HSCT, hematopoietic stem cell transplantation. OS, overall survival. PFS, progression-free survival. HR, hazard ratio. 95% CI, 95% confidence interval.

**Figure 2:**
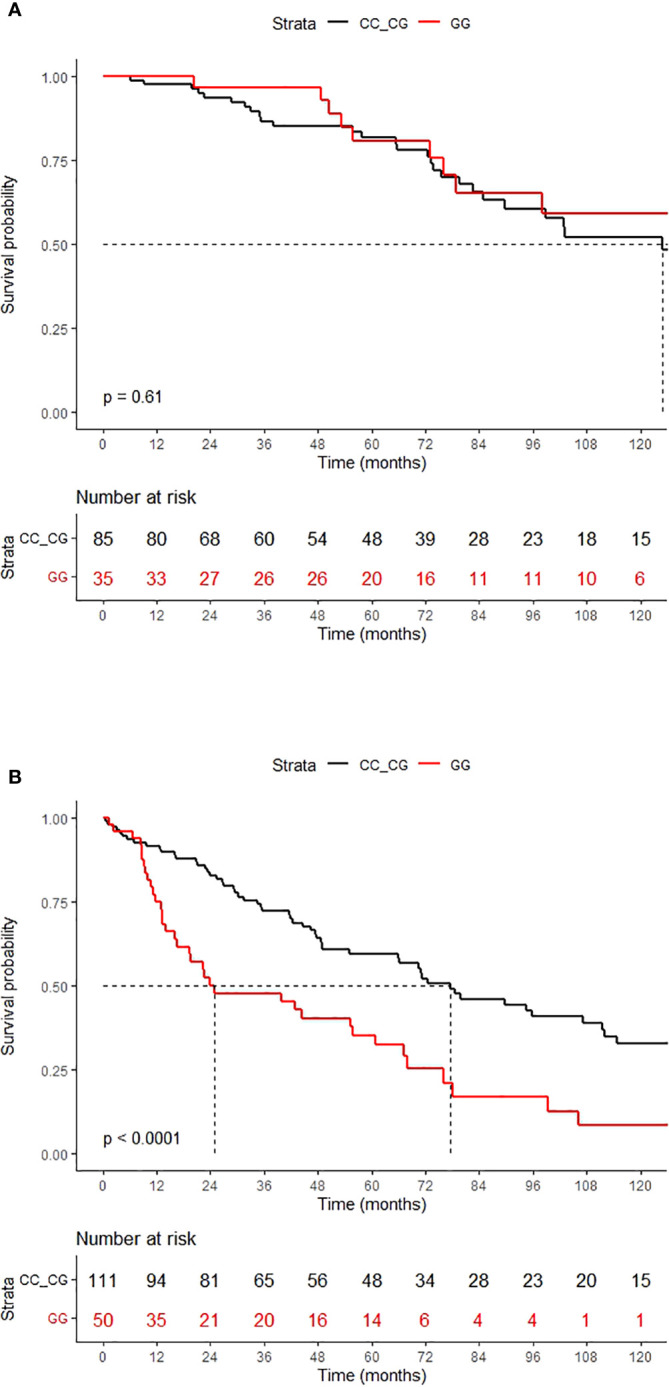
OS according to the CD200 rs1131199 genotype for patients who received HSCT **(A)** or did not **(B)**.

Subgroup analysis showed that the negative effect of the CD200 rs1131199 GG genotype was abolished in patients receiving an autologous stem cell transplant (auto-SCT): 5-years overall survival was 80.8% for patients with the CD200 rs1131199 GG genotype and 81.7% for the other genotypes (p: 0.6). However, the GG genotype was associated with a significantly lower median OS than those with CC+CG genotype when considering only patients who did not receive an auto-SCT: 24.8 months (95% CI: 16.6 – 67.1) *vs*. 77.5 months (95% CI: 65.9-111.7), respectively (p < 0.001) (**Figure 2**).

**Figure 3:**
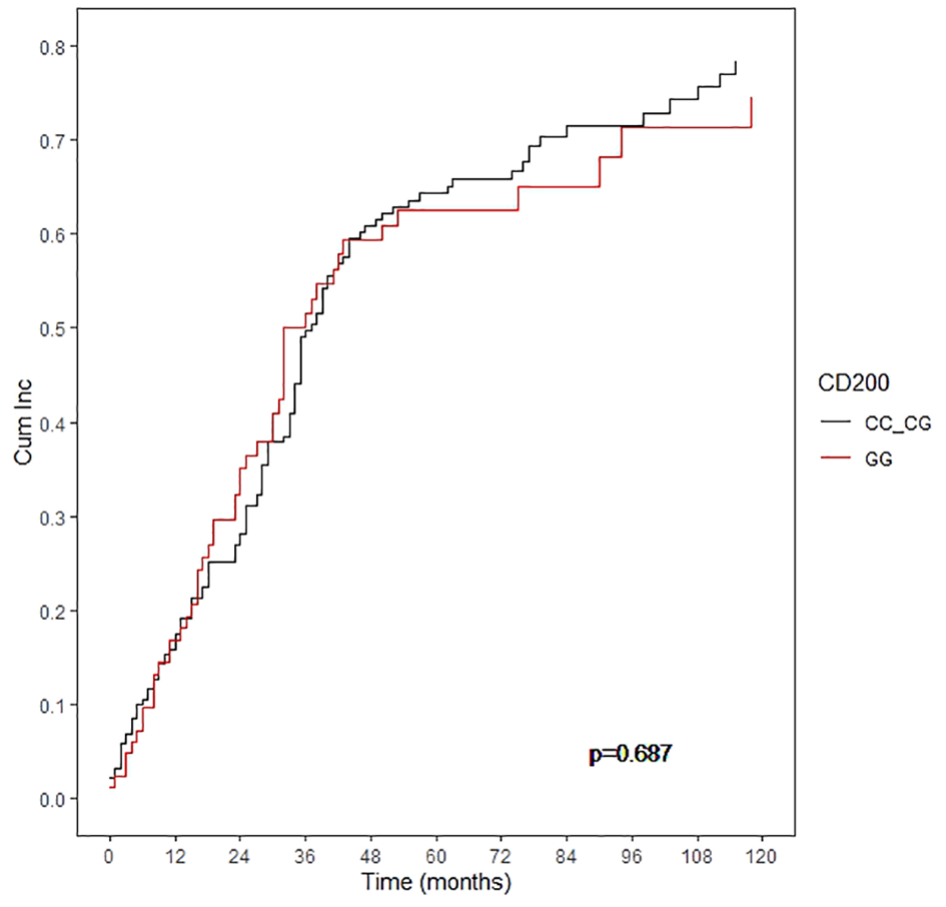
Cumulative incidence of progression according to the CD200 rs1131199.

Interestingly, the cumulative incidence of MM progression after first-line treatment was not different between genetic groups when considering the whole cohort at 5 years (59.8% for patients with the CD200 rs1131199 GG genotype and 66.3% for patients with CC or CG genotype; p: 0.687) (**Figure 3**). Consequently, Progression-free survival (PFS) was also not statistically different between patients homozygous for the G allele and those with rs1131199 CC or CG genotype (23.0% *vs* 27.9%; p: 0.42) at 5 years. This lack of correlation was maintained when considering only patients not receiving an auto-SCT, both for cumulative incidence of MM progression (p: 0.27) or for PFS (p: 0.063).

We intended to determine the reasons associated with the poor OS observed in patients with the CD200 rs1131199 GG genotype not receiving an auto-SCT (164 patients), and we define two different situations: a) patients who died after documentation of MM progression (death associated with disease progression, n:75) or b) patients dying without MM progression before dead (n:26).

**Figure 4:**
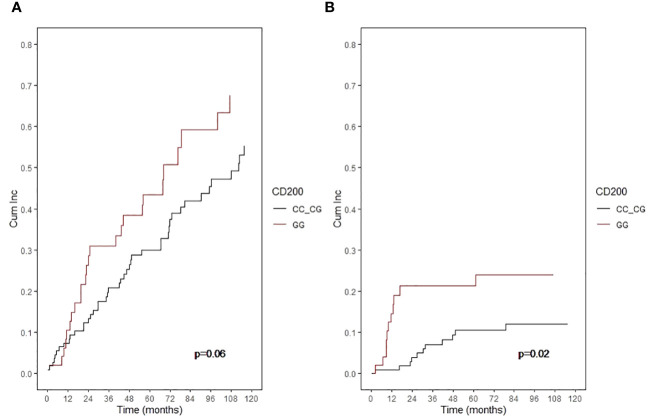
Cumulative incidence of mortality in patients not receiving an auto-SCT according to CD200 rs1131199 genotype for patients who died after documentation of MM progression **(A)** or without MM progression before dead **(B)**.

In patients not receiving an auto-SCT that presented MM progression, cumulative incidence of mortality was higher in patients with the CD200 rs1131199 GG genotype than in the CC or CG genotypes, but this difference did not reach statistical significance (p= 0.06) (**Figure 4A**). However, the multivariate analysis showed that the CD200 rs1131199 GG genotype was as an independent risk factor for higher mortality after MM progression (p = 0.007; HR: 2.1; 95% CI 1.25-3.52). High-risk cytogenetics (p = 0.036; HR: 3.7; 95% CI 1.5-9.2) and high ISS (p < 0.001; HR: 2.8; 95% CI 1.2-6.4) were also risk factors for higher mortality after detection of MM progression.

Moreover, when considering cumulative incidence of mortality without previously documented MM progression in patients not receiving an auto-SCT, the differences between genetic groups were even more evident: 21.2% of patients with CD200 rs1131199 GG genotype *vs* 10.5% for patients with pooled CC or CG genotypes (p: 0.02) (**Figure 4B**). As MM progression was not present in this group of patients, most of these patients died due to infection, toxicity, or organ failure. When analyzing the causes of death within this group, we found that 71.4% of patients with GG genotype died of infection (5 out of 12 patients), whereas infection was the final event in only 28.6% of patients with CG+GG genotype (2 out of 14 patients).

We did not find any significant association between the other analyzed CD200 polymorphism (rs2272022) and overall survival (AA+AC: 94.4 months *vs* CC: 72.8 months; p: 0.27) or progression-free survival (AA+AC: 29.9 months *vs* CC: 33.4 months; p: 0.97).

## Discussion

Immune dysfunction in MM plays an important role in disease pathogenesis and progression. Neoplastic evasion is facilitated by multiple cytokine and cellular signaling pathways, which decrease immune effector cell function (2). Increased expression of inhibitory immune checkpoint molecules such as programmed cell death ligand 1 (PDL-1) by myeloma and bone marrow microenvironment cells together with an increased expression of PDCD1 and CTLA4 on tumor infiltrating T cells contribute to maintaining the immunosuppressive state in MM (29, 30). CD200 protein acts as an inhibitory immune checkpoint molecule generating an immunosuppressive response in the microenvironment of several cancers including MM (12–14). Several studies have correlated high levels of CD200 expression in myeloma cells with worse prognostic (26–28). On the other hand, there is increasing evidence that some polymorphisms of inhibitory immune checkpoint gens are associated with risk of MM development or are associated with worse prognosis in patients with MM (20–24). We explored in previous study the association between genetic variants of the immune checkpoint molecules and survival in patients with MM. We analyzed the polymorphisms of CTLA4, BTLA, CD28, PD-1 and LAG-3 genes. Our results showed that the CTLA4 genotype identify patients with earlier progression of MM (25). Based on these interesting findings we decided explored other immune checkpoint molecule as CD200 in the same population.

There is currently no evidence regarding the relevance of genetic variants of CD200 gen in the clinical outcome of patients with MM. This is the first study that show an association between the CD200 genotype and overall survival in patients with MM.

The human CD200 gene is located in close proximity to those encoding CD80/CD86, on the long arm of chromosome 3 (3q13.2). This gene encodes a type I membrane glycoprotein containing two extracellular immunoglobulin domains, a transmembrane and a cytoplasmic domain (6). Expression of CD200 in T cells, both at the mRNA and protein level, is regulated by TNF-alpha and IFN-gamma (12).

In the present study, the rs1131199 CD200 GG genotype was associated with lower OS compared with the grouped CC and CG genotype in patients who have not received an auto-SCT. The rs1131199 polymorphism leads to an amino acid change from serine to cysteine at the codon 11 in the leader peptide (Ser11Cys). The biological effects of this substitution are currently unknown.

Interestingly, we did no find differences in cumulative incidence of progression and PFS as it has been previously described for CTLA4 genetic variants (25). These findings suggest that the studied genotypes of rs1131199 CD200 are not related with the mechanism of escape of cancer cells from immune surveillance control. Surprisingly, the rs1131199 GG genotype negatively impacted in the mortality not related with the progression of MM. This observation suggests that the GG genotype results in inadequate immune response leading to an increased risk of infection or a worse clinical outcome. In this direction, some studies suggest that CD200-CD200R interactions negatively regulate Toll-like receptor (TLR) signaling and reduce the production of pro-inflammatory cytokines by macrophage populations in the lung, in particular alveolar macrophages, which is essential for maintaining lung immune homeostasis. Importantly, during infection this interaction may lead to desensitization to bacterial TLR ligands, reducing chemokine production and NF-κβ activation on alveolar macrophages, thereby contributing to increased bacterial susceptibility (31, 32).

Several clinical trials have investigated the clinical benefit of checkpoint inhibitors in MM suggesting limited efficacy and significant toxicity of this approach. Early clinical trials targeting PD-1 have been discouraging (33). Pembrolizumab immunotherapy did not show objective response in MM, and its combination with lenalidomide, or pomalidomide in relapsed or refractory MM patients was associated with immune-related toxicities and mortality (34, 35). While PD-1 blockage has not demonstrated clinical benefits in MM patients, it is notable that some patients achieved long-term remissions after stopping pembrolizumab in clinical trials (36). Moreover, a phase 1 study of nivolumab in combination with ipilimumab for relapsed or refractory hematologic malignancies did not demonstrate favorable results in MM (37). TIGIT has emerged as an alternative strategy to checkpoint blockade, recent studies have demonstrated that blocking TIGIT using monoclonal antibodies promotes the effector function of MM patient CD8+ T cells. Accordingly, TIGIT monoclonal antibodies have been shown to prolong survival in preclinical MM models (18, 38).

Blockade of CD200 immune checkpoint has also been explored in a clinical trial phase 1 with samalizumab, a recombinant humanized monoclonal antibody that targets CD200, in relapsed or refractory B-cell chronic lymphocytic leukemia and multiple myeloma. The study showed some efficacy of samalizumab in a majority of patients with advanced B-CLL (64% of the total CLL), but progressive disease was observe in all patients with MM. The maximum tolerated dose was not determined and the severity of any reported adverse events related to samalizumab treatment was only mild to moderate. Although the clinical trial was discontinued due to administrative reasons, the preliminary findings from the study demonstrate the relative safety of samalizumab and its potential to reduce the tumor load associated with CLL (39). Recently, Shao A. et al. (40) reported a review about the CD200 expression and function in the tumor microenvironment as well as alternative strategies for potential neutralization of CD200 in human cancers, indicating that a probable explanation for the observed shortcomings in the samalizumab phase I trial could be the alternative mechanisms for the CD200 pro-tumorigenic role, beyond direct suppression of anti-tumor T cell responses, such as engagement of the CD200-CD200R axis, transcriptional mechanisms related to the cleaved cytoplasmic tail and ectodomain shedding. Consistently with these observations, combined blocking other immune checkpoint molecules such as CTLA-4 or PD-1 in addition to CD200 blockade could be an option to synergistically enhance antitumor activity and improve outcomes. Our results suggest that patients with CD200 rs1131199 GG genotype would improve their survival with a strict infection surveillance. It is unknown if blocking CD200 would restore or enhance immune response, decreasing the mortality not related with the progression of the disease.

Our results support the hypothesis that this CD200 polymorphism could be used as genetic marker to predict early mortality. The identification of personalized genetic biomarker profiles could improve the development therapies to improve MM prognosis. Clinical trials are ongoing to evaluate strategies that may enhance immune response by directly promoting T cell activity against myeloma cells, including immune checkpoint inhibitors, bispecific T-cell engagers and chimeric antigen receptor T cells. According to our results patients with CD200 rs1131199 GG genotype may be ideal candidates for inclusion in clinical trials exploring immune-based therapies, to analyze the clinical impact of combined immunotherapies with the aim of decreasing the early mortality due to infections and to progression of the disease. Further studies are needed to clarify the significance of immune genes variations and to determine whether genetic data could be used as predictive marker of survival.

## Data availability statement

The original contributions presented in the study are included in the article/**Supplementary Material**, further inquiries can be directed to the corresponding author/s.

## Ethics statement

The studies involving humans were approved by IDIBGI Biobank (Biobanc IDIBGI, B.0000872), 110 integrated in the Spanish National Biobanks Network. The studies were conducted in accordance with the local legislation and institutional requirements. The participants provided their written informed consent to participate in this study.

## Author contributions

YG-M conceived and designed the study. RR-R, AV, GO-G, MG-B and FL participated in data collection. AV and GO-G participated in data analysis. DG participated in manuscript drafting. All authors revised the manuscript critically for important intellectual content, gave their final approval of the version to be published and agreed to be accountable for all aspects of the work in ensuring that questions related to the accuracy or integrity of any part of the work are appropriately investigated and resolved.
