# Supplementary material for: CD200 genotype is associated with clinical outcome of patients with multiple myeloma
Source: Front Immunol. 2024 Feb 22;15:1252445. doi: 10.3389/fimmu.2024.1252445 (PMC10917927; doi:10.3389/fimmu.2024.1252445)
Supplement: Supplementary file 1 [file Table_1.docx]

**Table S1.** Clinical characteristics of smouldering MM patients according to CD200 rs1131199 and CD200 rs2272022.

| **Characteristics** | **Total** |  | **CD200 rs1131199** |  |  |  | **CD200 rs2272022** |  |  |
| --- | --- | --- | --- | --- | --- | --- | --- | --- | --- |
|  | ***%*** |  | **CC+CG** | **GG** | ***P*** |  | **AA+AC** | **CC** | ***P*** |
| Total | 67 (100.0) |  | 52 (77.6) | 15 (22.4) |  |  | 44 (69.8) | 19 (30.2) |  |
| Age (years) |  |  |  |  |  |  |  |  |  |
| Median (range) | 66 (59-72) |  | 67 (59-72) | 64 (56-70) | 0.248 |  | 67.5 (60.5-72.3) | 64 (56-69.5) | 0.245 |
|  |  |  |  |  |  |  |  |  |  |
| Sex |  |  |  |  |  |  |  |  |  |
| Men | 53.7 |  | 51.9 | 60.0 | 0.580 |  | 52.3 | 57.9 | 0.681 |
| Women | 46.3 |  | 48.1 | 40.0 |  |  | 47.7 | 42.1 |  |
|  |  |  |  |  |  |  |  |  |  |
| Age groups |  |  |  |  |  |  |  |  |  |
| <= 66 years | 50.7 |  | 48.1 | 60.0 | 0.416 |  | 45.5 | 63.2 | 0.197 |
| >66 years | 49.3 |  | 51.9 | 40.0 |  |  | 54.5 | 36.8 |  |
|  |  |  |  |  |  |  |  |  |  |
| Type of Monoclonal protein |  |  |  |  |  |  |  |  |  |
| IgG | 56.7 |  | 59.6 | 46.7 | 0.645 |  | 56.8 | 52.6 | - |
| IgA | 38.8 |  | 34.6 | 53.4 |  |  | 38.7 | 47.4 |  |
| Light chains | 3.0 |  | 3.8 | - |  |  | 2.3 | - |  |
| Others | 1.5 |  | 1.9 | - |  |  | 2.3 | - |  |
|  |  |  |  |  |  |  |  |  |  |
| History of MGUS | 24.2 |  | 28.8 | 7.1 | 0.093 |  | 25.0 | 21.1 | 0.736 |
|  |  |  |  |  |  |  |  |  |  |
| Cytogenetic profile (N=42) |  |  |  |  |  |  |  |  |  |
| High-risk cytogenetic by FISH* | 7.1 |  | 3.2 | 18.2 | 0.098 |  | 3.7 | 18.2 | 0.133 |
| Standard risk | 92.9 |  | 96.8 | 81.8 |  |  | 96.3 | 81.8 |  |
|  |  |  |  |  |  |  |  |  |  |
| LDH |  |  |  |  |  |  |  |  |  |
| High | 4.3 |  | 2.9 | 7.7 | 0.410 |  | 6.9 | - | 0.298 |
| Normal | 95.7 |  | 97.1 | 92.3 |  |  | 93.1 | 100.0 |  |

MM: multiple myeloma. MGUS: monoclonal gammopathy of undetermined significance. *FISH: del(17p), t(4;14), t(14;16). LDH: lactate dehydrogenase.
